# Curcumin-Loaded Micelles Dispersed in Ureasil-Polyether Materials for a Novel Sustained-Release Formulation

**DOI:** 10.3390/pharmaceutics13050675

**Published:** 2021-05-08

**Authors:** Kammila Martins Nicolau Costa, Mariana Rillo Sato, Tellys Lins Almeida Barbosa, Meiry Gláucia Freire Rodrigues, Ana Cláudia Dantas Medeiros, Bolívar Ponciano Goulart de Lima Damasceno, João Augusto Oshiro-Júnior

**Affiliations:** 1 Pharmaceutical Sciences Postgraduate Program, Center for Biological and Health Sciences, State University of Paraíba, Av. Juvêncio Arruda, S/N, Campina Grande 58429-600, Paraíba, Brazil; kammilamartiins@hotmail.com (K.M.N.C.); anaclaudia@uepb.edu.br (A.C.D.M.); bolivarpgld@servidor.uepb.edu.br (B.P.G.d.L.D.); 2 School of Pharmaceutical Sciences, São Paulo State University (UNESP), Araraquara 14800-903, São Paulo, Brazil; mari_sato_@hotmail.com; 3 Postgraduate Program in Chemical Engineering, Federal University of Campina Grande, Aprígio Veloso, 882, Campina Grande 58429-970, Paraíba, Brazil; tellyslins@hotmail.com (T.L.A.B.); meirygfr@hotmail.com (M.G.F.R.); 4 UNIFACISA University Center, Manoel Cardoso Palhano, Campina Grande 58408-326, Paraíba, Brazil

**Keywords:** *Candida albicans*, technological innovation, nanostructured dispersed

## Abstract

Vulvovaginal candidiasis (VVC) is a vulvar/vaginal infection that affects approximately 75% of women worldwide. The current treatment consists of antimicrobials with hepatotoxic properties and high drug interaction probabilities. Therefore, this study aimed to develop a new treatment to VVC based on micelles containing curcumin (CUR) dispersed in a ureasil-polyether (U-PEO) hybrid. The physical-chemical characterization was carried out in order to observe size, shape, crystallinity degree and particle dispersion in the formulation and was performed by dynamic light scattering (DLS), scanning electron microscopy (SEM), X-ray diffraction (XRD) and through in vitro release study. The results of DLS and SEM exhibited micelles with 35 nm, and encapsulation efficiency (EE) results demonstrated 100% of EE to CUR dispersed in the U-PEO, which was confirmed by the DRX. The release results showed that CUR loaded in U-PEO is 70% released after 10 days, which demonstrates the potential application of this material in different pharmaceutical forms (ovules and rings), and the possibility of multidose based on a single application, suggesting a higher rate of adherence.

## 1. Introduction

Vulvovaginal candidiasis (VVC) is conceptualized as an infection of the vulva and vagina, with occurrences of intense vulvar itching, leukorrhea, dyspareunia, dysuria, edema and vulvovaginal erythema, in which itching is the most decisive symptom when comparing VVC to vulvovaginitis of another etiology [1]. VVC affects approximately 75% of women around the world at least once in their lifetime, where 50% present a recurrence within one year. Yeasts of the genus *Candida*, mainly of the species *Candida albicans*, are considered the main fungus that causes VVC (about 85%) [2,3].

The treatment of choice for VVC employs the use of several antifungals. Among them are the amphotericin B, nystatin and azole drugs, which represent the class with the highest number of active substances, such as fluconazole, ketoconazole, butoconazole, itraconazole and miconazole, which are commonly used in suspension form, applied topically over the lesion, or in the form of tablets and ointments [4]. However, these drugs have a prolonged treatment time, portray side effects such as hepatotoxicity and perform drug interactions, which can aggravate the condition. Additionally, studies have shown that the organization of microorganisms in biofilm promotes a considerable decrease in susceptibility to antimicrobial agents, inhibiting the drugs’ actions and increasing resistance to the aforementioned drugs [5,6].

In this context, innovative research focuses on the design and development of alternative treatments, including the use of photodynamic therapy (PDT) [7,8,9]. PDT is based on the principle that a photosensitizer binds to the target cell and is activated by a light in a specific wavelength. In this reaction, reactive oxygen species are obtained and are extremely toxic to microorganisms, consequently leading to their deaths and causing low damage to the host since the light is radiating into the pathogen’s pharmacological target [10,11,12,13].

Curcumin (CUR), a natural dye derived from *Curcuma longa* L. that possesses antifungal and antibacterial activity, has been studied as a photosensitizing agent in PDT and can stimulate immunomodulation of the host’s inflammatory response, improving the healing process [14,15,16]. Several studies showed the efficacy of CUR against pathogens and in association with PDT, in which the effects become more satisfactory against fungi and bacteria resistant to conventional drugs [9,17].

However, CUR is a compound with hydrophobic characteristics and may undergo rapid degradation in the presence of light and in an aqueous medium. Due to the toxic effects presented by organic solvents, ethanol and dimethyl sulfoxide (DMSO) [18,19], a variety of nanostructured systems for controlled release have been developed to improve the bioavailability, solubility and therapeutic efficacy of CUR [20,21,22,23,24,25,26,27,28,29,30]. Nanosystems have been developed to circumvent CUR solubility, such as polymeric nanoparticles, inorganic nanoparticles and liposomes; however, micelles have several advantages such as the ability to incorporate lipo and water-soluble drugs, low cost and ease of large-scale production [31,32,33,34,35,36].

Micelles are colloidal-sized particles (10–100 nm) composed of an amphiphilic polymer that after a certain concentration are acknowledged as critical micellar concentrations (CMC) and start to aggregate in spheres where all the hydrophilic ends turn outwards, creating a hydrophobic environment inside that interacts with the active compound, such as curcumin, by hydrogen bonds and/or hydrophobic interactions, thereby allowing its encapsulation, hence improving solubility [28,29,30]. Recently, our group showed CUR-micelles’ activity against *S. mutans* and *C. albicans* biofilms in a low concentration of 270 µM [37].

Nevertheless, micelles are very fluid, presenting a liquid character, and may cause rapid dispersion when administered into the vagina or oral mucosa. Therefore, dispersion in ureasil-polyether (U-PEO) material can be an attractive tool to overcome this limitation.

These U-PEO hybrid materials synergistically combine the physical and chemical characteristics of their components, providing unique properties and making this class of materials excellent for the development of new multifunctional systems with an extensive possibility of applications. The organic phase provides specific physical or chemical properties (optical, electrical, reactivity), while the inorganic phase increases mechanical resistance, thermal stability and allows modulation of the refractive index. In addition, the rheological properties favor the processing of the final material with the possibility of varying shapes and sizes [38,39,40,41].

Therefore, such properties are interesting due to the ease of preparation and the possibility of optimizing the time of contact of the final formulation with the vaginal mucosa or other tissues (oral and epidermal) [42,43]. Moreover, the uptake and release of CUR from micelles has already been reported in the literature without scale-up success. As such, here we have incorporated CUR into micelles, added it into U-PEO material and analyzed its release profile to provide a concrete option in a pharmaceutical form (ovules/rings vaginal or film). The obtained results provide an insight into the possibility of multidose based on a single application, suggesting a higher rate of adherence.

## 2. Materials and Methods

### 2.1. Materials

Curcumin (CUR); Pluronic^®^ F-127 (P127); 3-isocyanatopropyltriethoxysilane and modified polymers (NH_2_-POE-NH_2_) were obtained from Sigma-Aldrich (São Paulo, Brazil).

### 2.2. Preparation of Polymeric Micelles

The methodology used to obtain the polymeric micelles was based on the solvent evaporation technique or “polymeric film”, with adaptations. First, 1.0 g Pluronic^®^ F-127 (P127) was weighed and transferred to a 50 mL round-bottom flask. This amount was solubilized in 25 mL of chloroform, which was evaporated with the assistance of an Ika^®^ RV 8 evaporator for 10 min at 45 ℃. The polymeric film formulation was rehydrated with exactly 30 mL of purified water and subsequently taken to the magnetic stirrer (SL-92 SOLAB) for 30 min with an agitation of 700 rpm. The amount of 1.0 g of the copolymer in 30 mL of water is based on its critical micellar concentration (2.8 × 10^−6^ M) [44].

In the polymeric film formation stage, different percentages (0.1, 0.5, 1.0 and 3.0) of CUR, in relation to the polymer mass, were added in order to evaluate the maximum drug concentration solubilized in the micelles. After the incorporation of CUR in the micelles, the formulation was taken to the ultrasonic cell switch, also called ultrasonic homogenizer equipment (UNIQUE^®^, São Paulo, Brazil), with an amplitude of 35% for 1 min.

### 2.3. Physico-Chemical Characterization of CUR-Loaded Micelles

#### 2.3.1. Determination of the Average Hydrodynamic Diameter (d nm)

The mean diameter analysis of the CUR-loaded micelles was performed after the development of the micelles using the light scattering technique of the hydrodynamic ray or dynamic light scattering (DLS) of the suspended particles using Nanotrac equipment (Nanotrac Wave Model MN401, São Paulo, Brazil). The measurements were performed in triplicate with a temperature of 25 °C, and the samples were diluted in the proportion of 1:30 (*v/v*) in distilled water.

#### 2.3.2. Scanning Electron Microscopy (SEM)

To analyze the particle size and shape of micelles with or without CUR and to provide information on size difference with the presence of CUR, the technique used was high-resolution scanning electron microscopy (SEM-FEG) with JEOL JSM-7500F equipment. Subsequently, a drop of the material was placed on a metallic support and dried at room temperature for three days in a desiccator. The support was then coated with a conductive material (carbon), and photomicroscopy (100,000× magnification) was obtained.

#### 2.3.3. Encapsulation Efficiency (EE%)

The encapsulation efficiency of the micelles containing CUR was performed after centrifugation at 5000 rpm for 30 min. Removing the supernatant, UV-Vis was quantified so that the encapsulation efficiency (EE) was determined using the line equation, determined from the linear regression.

The linear regression was determined using the UV-Vis Spectrophotometer SHIMADZU UV-1900 (São Paulo, Brazil) in a 426 nm wavelength. The medium used to carry out this step was composed of an acetate buffer pH 4.0 (80%) as a means of replicating the vaginal environment, Tween® 80 (15%) and absolute ethanol (5%), to guarantee the sink condition.

From a stock solution, composed of the added medium and CUR, at a concentration of 100 µg/mL, dilutions were prepared at concentrations of 0.5, 1, 2, 4, 6 and 10 µg/mL. The evaluations were performed in sextuple by different analysts, on alternating days and analyzed in Excel.

### 2.4. Preparation of Ureasil-Polyether Hybrid Materials

The precursors were obtained from the reaction of a modified alkoxide 3-isocyanatopropyltriethoxysilane (IsoTrEOS) and modified polymers (NH_2_-POE-NH_2_) with molecular masses of 500 g/mol. The mixture of alkoxide and modified polymer was kept under reflux in absolute ethanol at a temperature of 80 °C for 24 h. Subsequently, the synthesis solvent was eliminated by heating and reduced pressure [45].

The hydrolysis and condensation reactions were then promoted by the precise addition of 0.750 g of the precursor, 50 µL of purified water, 500 µL of ethanol, 100 µL of an acid catalyst agent (HCl 2 M) and agitation of 700 rpm in a magnetic stirrer for 1 min, leading to gel formation. The micelles containing 0.1, 0.5, 1.0 and 3.0% of CUR were incorporated in this step of the hydrolysis and condensation. Subsequently, the U-PEO remained in a desiccator to dry at room temperature (±30 °C) for 72 h.

### 2.5. X-ray Diffraction (XRD)

The diffractograms were obtained with the XRD 6000 X-ray diffractometer model (Shimadzu^®^,São Paulo, Brazil) with angular scanning 5° < 2θ < 35° in the Bragg–Brentano assembly, θ–2θ system, using Cu (kα1) radiation with scanning in step 0.02 (2θ) with an interval of 0.6 s for each sample.

### 2.6. In Vitro Release Assay

The in vitro release study was carried out using three groups: (i) U-PEO with micelle and CUR, (ii) U-PEO with CUR and (iii) pure CUR. These were immersed separately in a receiver solution composed of an acetate buffer pH 4.0, 15%, Tween® 80 and 5% absolute ethanol, in addition to a beaker containing 750 mL of it under agitation on a magnetic stirrer SL-92 SOLAB at 100 rpm at 37 °C. The entire system was protected from light. After the predetermined time intervals (15, 30, 45 min, 1, 2, 3, 4, 6, 12, 24, 48, 72, 96, 120, 144, 168, 192, 216 and 240 h), a 3.0 mL aliquot of the solution was collected and analyzed on the UV-vis spectrophotometer in the wavelength range of 426 nm. The experiment was carried out in triplicate.

## 3. Result and Discussion

### 3.1. Preparation of Polymeric Micelles with and without CUR

Micelles, with and without CUR, were prepared through the polymeric film method, which allows the incorporation of hydrophobic active substances in high concentrations. After this step, the material was hydrated and taken to the ultrasonic homogenizer, a high-energy method that allows the obtainment of particles in nanometric size.

The material obtained in the form of a white micelle, which does not contain CUR, was characterized as a fluid and translucent liquid, in which the Tyndall effect was observed. On the other hand, a characteristic light-yellow color was observed in the micelles that presented CUR, and it would darken gradually with the increase of the CUR concentration.

Visually, it was possible to determine that when the CUR concentration was higher than 1%, it was not entirely dispersed or solubilized in the micelles. This observation was acknowledged through the formation of precipitates or turbidity of the colloidal solution.

### 3.2. Determination of the Average Hydrodynamic Diameter (d nm) of Micelles

The results revealed that the micelles with CUR presented average values of size between 20 and 60 ± 3.50 nm (Figure S1), with a large part of the particle number size distributions at 35 nm, which is in agreement with studies carried out by Vaidya et al. (2018) [46], where the researchers obtained DLS results that, through the application of the same micelle preparation method, showed that all micelles loaded with CUR obtained a narrow size distribution, but similar to that found in this study, confirmed the efficiency of the method used to obtain the pure micelles loaded with CUR.

### 3.3. Scanning Electron Microscopy (SEM)

**Figure 1:**
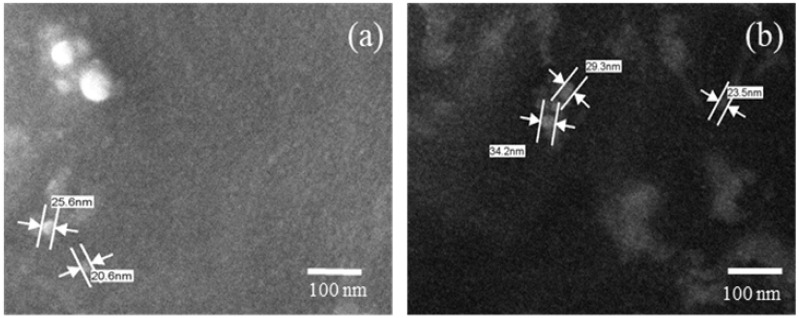
Photomicroscopy of the micelles: (**a**) micelle without curcumin and (**b**) micelle with curcumin, 100,000× magnification.

SEM is a technique used to analyze the particle size and morphology of micelles. Thus, the results of SEM show that the addition of CUR did not cause significant morphological alterations to the size and morphology of the micelles without CUR. The results showed spherical micelles with nanometric sizes between 20 and 34 nm (Figure 1). The modification of the hydrated state of the micelles from the SEM technique does not present a significant rate of change in the average particle size.

### 3.4. Encapsulation Efficiency (EE)

The linearity of the method for quantifying the encapsulation efficiency and CUR release by spectroscopy in the UV region in the absorbance range of 426 nm was determined by constructing a linear regression using a CUR concentration ranging between 0.5 and 10 µg/mL

The equation of the generated line was y = 0.1186x + 0.0022, and the correlation coefficient (R) was 0.9999, which is linear, as shown in Figure S2. Thus, the linear regression data were subjected to an analysis of variance (lack of fit and significance of the regression) using the Snedecor F test to assess the fit of the model. The results revealed that in this model, there was no lack of adjustment (lack of adjustment obtained = 1.17 < Ft = 2.68) and it presented significant regression (~9500 times), validating the model given by the line equation.

The residual graph (Figure S3) allowed the verification of the behavior of the data variances in the linear regression in relation to the concentration increase, which is homoscedastic. In addition, the limit of detection and the limit of quantification were determined using the parameters of the linear regression, obtaining results of 0.010 and 0.032, respectively.

Therefore, micelles with different CUR concentrations were analyzed after the centrifugation and filtration processes. The results revealed that the EE for micelles containing 0.1, 0.5, 1 and 3% of CUR were 96.2 ± 3.5, 86.4 ± 5.7, 62.9 ± 2.2 and 20, 2 ± 0.7%, respectively. Results that agree with the visual analysis are where, after 0.5% of CUR, the colloidal solution presented agglomerates/precipitates or cloudy appearance. In the result presented by Gong et al. (2013), the EE of the CUR incorporated into the micelles was 98.40%, using similar proportions [47], as well as the presented particle size, which was 26.9 nm.

### 3.5. Obtainment of Ureasil-Polyether Hybrid Membranes

The films composed of the U-PEO are formed by the sol-gel process, which involves hydrolysis and condensation reactions. The speed of these reactions (film formation time) is controlled by the variation of the precursor/catalyst ratio [38,39,40,41].

**Figure 2:**
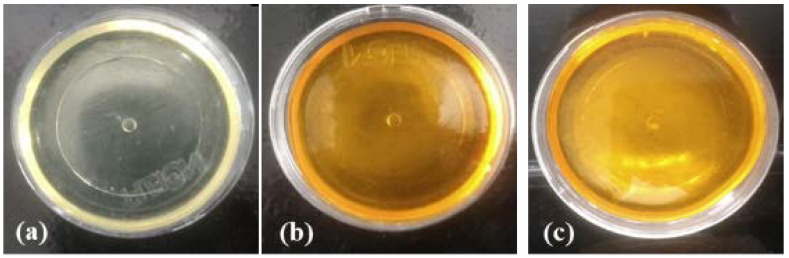
Pure ureasil-polyether (U-PEO 500) hybrid material (**a**) with curcumin (**b**) and curcumin-micelle 3% (**c**).

In the final result, after the drying process, the material must appear homogeneous and present flexibility. For this reason, its obtainment must be carried out methodically, considering that factors such as the pressure and the temperature of the environment can interfere, resulting in cracks and brittle materials. Figure 2 shows the visual characteristics of the developed U-PEO containing the micelles with and without CUR.

The visual results reveal that the presence of the drug and the micelles, in different concentrations, in the membrane did not alter the material’s organoleptic properties. In addition, it is possible to observe that, when incorporated into the U-PEO, the micelle containing 3% of CUR is visually homogeneous, without the presence of agglomerates or precipitates. The U-PEO, due to its distinct organic groups (OH, C-O-C and N-H), is able to assist in the solubilization of different molecules (hydrophilic, hydrophobic and metals) [48].

### 3.6. X-ray Diffraction (XRD)

**Figure 3:**
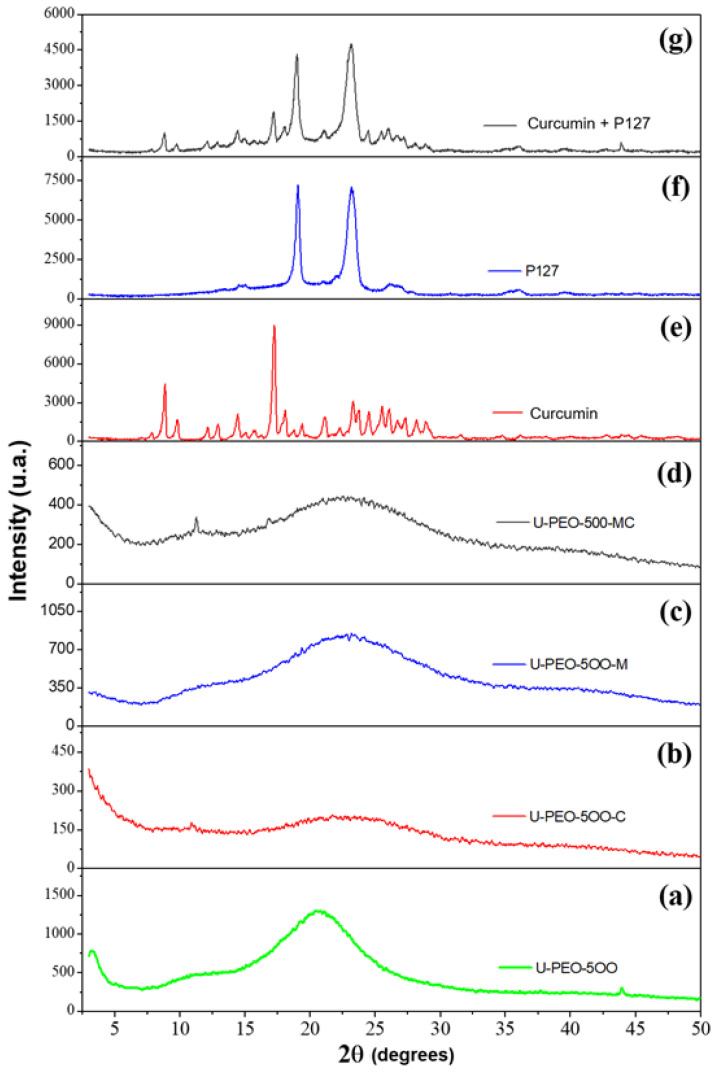
Sample XRD patterns: (**a**) pure U-PEO, (**b**) U-PEO + curcumin, (**c**) U-PEO + pure micelle, (**d**) U-PEO + micelle + curcumin, (**e**) pure curcumin, (**f**) pure micelle and (**g**) curcumin + micelle.

Figure 3 shows the XRD patterns of the samples of the U-PEO, U-PEO containing CUR, U-PEO containing the micelle singly, U-PEO containing micelles with CUR, only CUR, only micelles and micelles containing CUR.

In the XRD analysis results of the pure CUR, it is possible to observe its crystalline form with characteristic peaks, which are more evident in the range of 16°. Sun et al. (2013) [49] mention in their research that pure CUR exists in a crystalline state, exhibiting several characteristic reflections between 10 and 30° 2θ. The P127 presented two characteristic points in the range of 19° and 23°, remaining unchanged even after the incorporation of CUR in the micelles, similarly as it happens in other works, as in the research carried out by Sahu et al. (2011), where the results were similar to those presented in this study [50,51].

The X-ray of the materials synthesized from the U-PEO, CUR, and micelles (P127) demonstrate an amorphous pattern and the disappearance of the CUR and the P127 peaks, suggesting dispersion of both in the material, hence corroborating the visual characteristics.

### 3.7. In Vitro Release Assay

**Figure 4:**
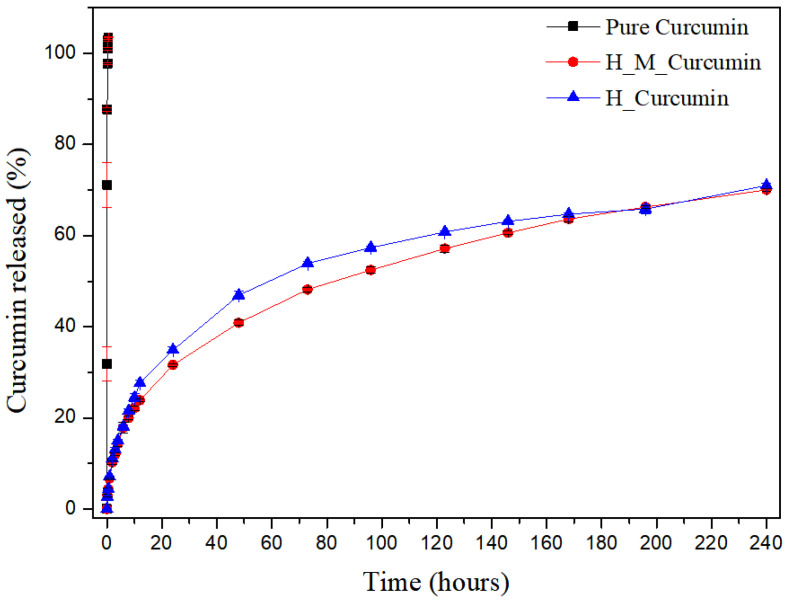
Release of curcumin present in a U-PEO material as a function of time. Accumulative release of curcumin as a function of time from pure curcumin (black line), U-PEO + curcumin (blue line) and U-PEO + micelle + curcumin (red line).

Figure 4 shows the release profile of pure CUR (black), U-PEO with CUR (blue), and U-PEO with micelles and CUR (red). It is possible to observe that CUR reached 100% release in approximately 15 min. The U-PEO with pure CUR and the U-PEO with micelles and CUR presented a different profile, in which CUR had a release rate of around 40% in the first 48 h and thereafter, every 24 h a rate of 5% is released until the 10th, reaching a range of 70% release of CUR, featuring a controlled and prolonged release. These results demonstrate that a developed system can be used in prolonging treatment, reducing the plasma fluctuations in CUR levels, which reduces side effects and increases patient compliance with treatment.

The micelles did not have a significant influence on the chemical bonds involving CUR and U-PEO. U-PEO’s intrinsic characteristics of having a hydrophobic-hydrophilic character affect its ability to swell and release the CUR along with the micelles. However, the advantage of the presence of the micelles in the system is due to the influence on the CUR solubility increase through its encapsulation in the micelles, since in a physiological environment its solubility is reduced and, consequently, its bioavailability as well.

In the literature, several nanostructured systems have been used to improve CUR release profiles, such as the research conducted by Anitha et al. (2011) [52], using nanoparticles (NPs) as a new release system. The authors mention that the release pattern of the drug showed a burst release in the first 3 h, followed by a controlled release of CUR over a period of one week, in which about 70% of the drug was released during that time. The CUR that is adsorbed on the surface of the NPs, and the drug trapped near the surface may be the reason for the initial burst release. Since the rate of dissolution of the polymer near the surface is high, the amount of drug released will also be high. For Sahu et al. (2011) [51], who used polymeric micelles as a source of a new release system, relatively quick release occurred in the first stage, followed by a sustained and slow release over a prolonged period of up to 10 days. During the first 4 h, there was a 5% release of the Pluronic F127 micelles. After that, the release was slow and sustained and, finally, at 240 h, 63% of the drug was released from the Pluronic F127 micelles.

## 4. Discussion and Conclusions

Based on conventional dosage forms (ointments, creams, and suspensions), which are ineffective due to the mechanisms of clearance of epithelial and mucous tissues, and in all scientific papers using liquid nanosystems containing CUR, that have not advanced to clinical studies due to their fluidity and lack of adherence in the target tissue, this work demonstrated the development of a new a pharmaceutical form capable of overcoming these drawbacks, such as the low solubility of CUR, no use of toxic solvents, such as DMSO and ethanol and faster dispersion of the micelles or any other nanostructured pharmaceutical form. Recently, our group showed the chosen CUR-micelles activity against *S. mutans* and *C. albicans* biofilms in a low concentration of 270 µM [37].

Our future projections are to seek partnerships to produce a pilot batch, aiming at the adaptation of the pharmaceutical form within the norms established by the main regulatory agencies for the physical-chemical and microbiological quality control, aiming at phase 1 clinical studies.

This research made it possible to conclude that the synthesis of the micelles containing CUR was carried out properly since the results of DLS and SEM corroborate the result of the encapsulation efficiency, which demonstrated that the incorporation of CUR did not change the hydrodynamic size properties of the micelles as well as the dispersion of micelles containing CUR in the ureasil-polyether material, since the XRD results reveal that the CUR remains soluble. In vitro release results demonstrate that this combination is capable of being a prolonged release system, where, after 10 days, approximately 70% of the CUR was released. These results demonstrate the potential of the systems to be a multidose treatment based on a single application, suggesting a higher rate of adherence and a lower rate of abandonment by the patient, in addition to providing the material for several pharmaceutical forms, such as eggs and vaginal rings, adapting to the patient’s needs. However, further clinical studies are needed to confirm these results.

## Data Availability

Link to publicly archived datasets analyzed or generated during the study. Please refer to suggested Data Availability. https://1drv.ms/u/s!AnTvCVJp6xpjhbI5B5uXkELCAUC4Ug?e=Txxcx2.

## Supplementary Materials

The following are available online at https://www.mdpi.com/article/10.3390/pharmaceutics13050675/s1, Figure S1: Particle size measurement of Micelles using DLS in water, Figure S2: Linear regression of CUR in pH 4.0 acetate buffer, Figure S3: Residue graph.
