# Supplementary material for: Curcumin-Loaded Micelles Dispersed in Ureasil-Polyether Materials for a Novel Sustained-Release Formulation
Source: Pharmaceutics. 2021 May 8;13(5):675. doi: 10.3390/pharmaceutics13050675 (PMC8151228; doi:10.3390/pharmaceutics13050675)
Supplement: Supplementary file 1 [file pharmaceutics-13-00675-s001.zip › pharmaceutics-1216851-supplementary.pdf]

pharmaceutics

MDPI

Supplement Materials: Curcumin-Loaded Micelles Dispersed in Ureasil-Polyether Materials for a Novel Sustained-Release Formulation

Kammila Martins Nicolau Costa, Mariana Rillo Sato, Tellys Lins Almeida Barbosa, Meiry Gláucia Freire Rodrigues, Ana Cláudia D. Medeiros, Bolívar Ponciano Goulart de Lima Damasceno and João Augusto Oshiro-Júnior

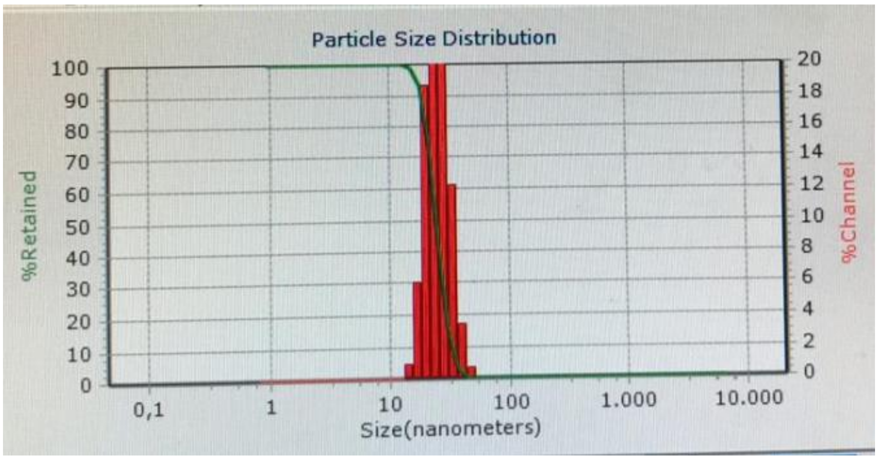

Figure S1. Particle size measurement of Micelles using DLS in water.

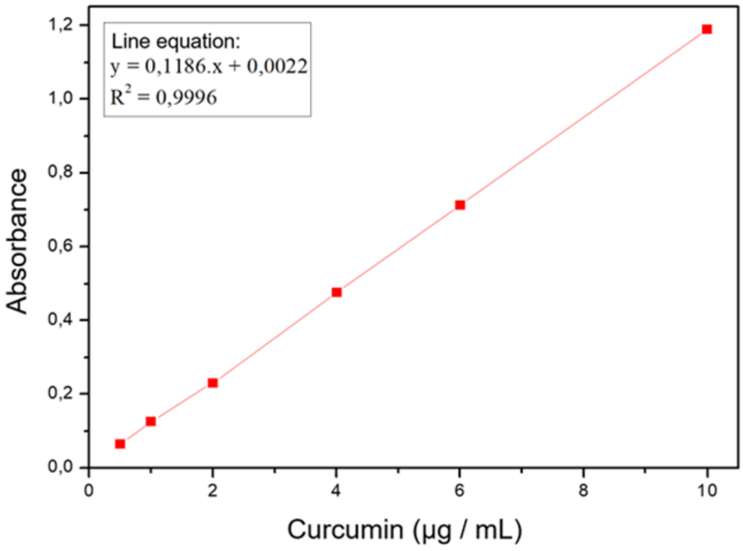

Figure S2. Linear regression of CUR in pH 4.0 acetate buffer.

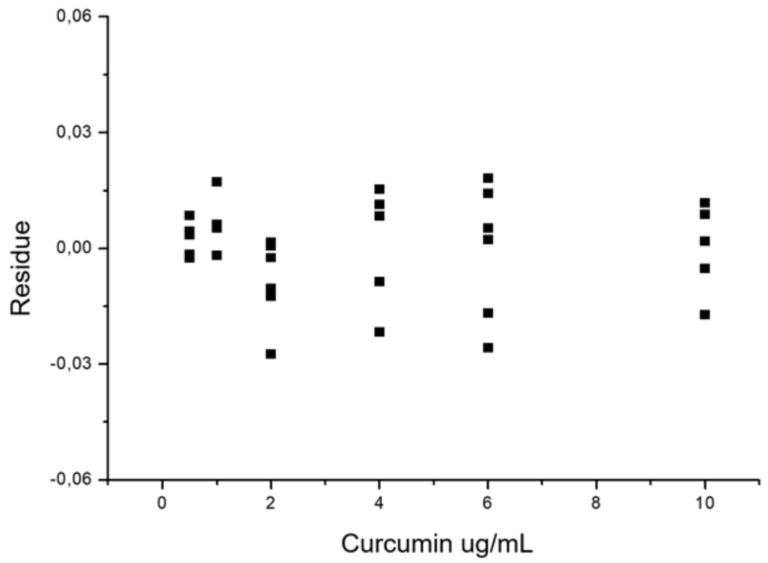

Figure S3. Residue graph.
